# Genetic Diversity of Newcastle Disease Virus Involved in the 2021 Outbreaks in Backyard Poultry Farms in Tanzania

**DOI:** 10.3390/vetsci10070477

**Published:** 2023-07-21

**Authors:** Charlie F. Amoia, Jean N. Hakizimana, Nisha K. Duggal, Augustino A. Chengula, Mohammed A. Rohaim, Muhammad Munir, James Weger-Lucarelli, Gerald Misinzo

**Affiliations:** 1 Department of Veterinary Microbiology, Parasitology and Biotechnology, Sokoine University of Agriculture, Morogoro 67125, Tanzania; 2 SACIDS Africa Centre of Excellence for Infectious Diseases, SACIDS Foundation for One Health, Sokoine University of Agriculture, Morogoro 67125, Tanzania; 3 Department of Biomedical Sciences and Pathobiology, Virginia-Maryland College of Veterinary Medicine, Virginia Tech, Blacksburg, VA 24060, USA; 4 Center for Emerging, Zoonotic, and Arthropod-Borne Pathogens, Virginia Tech, Blacksburg, VA 24060, USA; 5 Division of Biomedical and Life Sciences, Faculty of Health and Medicine, Lancaster University, Lancaster LA1 4YG, UK; 6 Department of Virology, Faculty of Veterinary Medicine, Cairo University, Giza 12211, Egypt

**Keywords:** newcastle disease virus (NDV), tanzania, East Africa, poultry, genotypes, phylogenetic analyses

## Abstract

**Simple Summary:**

The genetic nature of currently circulating Newcastle disease virus (NDV) genotypes in backyard poultry flocks in East Africa is poorly understood. Genetic characterization of the NDV fusion gene of field isolates collected from six regions (Arusha, Dar es Salaam, Dodoma, Iringa, Morogoro and Rukwa) of Tanzania during 2021 highlighted the spread of NDV genotype VII in Tanzanian backyard poultry. The class II subgenotype VII.2 of the virus has been identified as the currently dominant strain in all studied regions, followed by class II subgenotype XIII.1.1. Our findings provide foundational information on the genetic diversity of NDV in several regions of Tanzania, which is important for better design of vaccines and subsequent control of the disease in the country and the region.

**Abstract:**

Newcastle disease virus is a significant avian pathogen with the potential to decimate poultry populations all over the world and cause enormous economic losses. Distinct NDV genotypes are currently causing outbreaks worldwide. Due to the high genetic diversity of NDV, virulent strains that may result in a lack of vaccine protection are more likely to emerge and ultimately cause larger epidemics with massive economic losses. Thus, a more comprehensive understanding of the circulating NDV genotypes is critical to reduce Newcastle disease (ND) burden. In this study, NDV strains were isolated and characterized from backyard poultry farms from Tanzania, East Africa in 2021. Reverse-transcription polymerase chain reaction (RT-PCR) based on fusion (*F*) gene amplification was conducted on 79 cloacal or tracheal swabs collected from chickens during a suspected ND outbreak. Our results revealed that 50 samples out 79 (50/79; 63.3%) were NDV-positive. Sequencing and phylogenetic analyses of the selected NDV isolates showed that 39 isolates belonged to subgenotype VII.2 and only one isolate belonged to subgenotype XIII.1.1. Nucleotide sequences of the NDV *F* genes from Tanzania were closely related to recent NDV isolates circulating in southern Africa, suggesting that subgenotype VII.2 is the predominant subgenotype throughout Tanzania and southern Africa. Our data confirm the circulation of two NDV subgenotypes in Tanzania, providing important information to design genotype-matched vaccines and to aid ND surveillance. Furthermore, these results highlight the possibility of the spread and emergence of new NDV subgenotypes with the potential of causing future ND epizootics.

## 1. Introduction

Newcastle disease (ND) is the world’s most destructive poultry disease, despite comprehensive vaccination efforts [1]. Newcastle disease virus (NDV), the causative agent of ND, belongs to the family *Paramyxoviridae* and genus *Orthoavulavirus* [2], and has a non-segmented, negative-sense RNA genome that encodes for six proteins: nucleocapsid protein (NP), phosphoprotein (P), matrix protein (M), fusion protein (F), hemagglutinin–neuraminidase protein (HN), and large protein (L) [3]. RNA editing of the P messenger RNA produces two additional proteins: V and W [4]. The two surface glycoproteins, HN and F, play a major role in virus infection and are major targets of the host adaptive immune response [5]. Importantly, the presence of a multi-basic cleavage site in the F protein is a major virulence determinant of NDV [6]. Moreover, the *F* gene is commonly used for phylogenetic analyses because it is highly variable among different NDV strains, and it is also relatively conserved within a particular NDV genotype, making it a useful marker for distinguishing between different genotypes of the virus [7].

The NDV is categorized into two major classes. Class I viruses are typically avirulent and isolated from wild birds. Class II viruses include both avirulent and virulent isolates from wild birds and domestic poultry [8,9]. At least 21 genotypes (I to XXI) were recognized within class II [10]. The severity of disease caused by NDV differs widely between strains [11], and viruses are categorized for their virulence into three categories from least to most virulent: lentogenic (infection with subclinical to mild respiratory disease), mesogenic (symptoms of respiratory disease and nervous disorders with low mortality), or velogenic (hemorrhagic gastroenteritis, pneumonia, and/or encephalitis, deaths and high morbidities up to 100%) [12]. The disease brought on by velogenic NDV strains can affect chickens of any age, whereas only young birds are susceptible to disease caused by lentogenic and mesogenic strains [13,14].

Genotype VII is an increasingly common velogenic NDV strain that can be further subdivided into three subgenotypes: VII.1.1, VII.1.2 and VII.2 [15]. Subgenotypes VII.1.1 and VII.1.2 were responsible for the fourth NDV panzootic that occurred in Europe, Asia, and the Middle East in the 1990s, as well as subgenotype VII.2 that originated in Indonesia [16,17,18]. Several economically important outbreaks have been linked to genotype VII viruses since 1984, including those in Asia, Europe, Africa and some parts of America [8,19,20]. More knowledge about this genotype is needed, given its widespread distribution. In addition, genotype XIII has two subgenotypes, namely, subgenotypes XIII.1 and XIII.2. Genotype XIII is responsible for substantial economic losses in Southern, Western and Central Asia [18,21,22,23,24] and in Africa [25].

In Tanzania as well as many other African countries, ND causes significant poultry losses [26]. In Tanzania, where 86% of livestock-keeping households keep chickens as their primary livestock, ND is an endemic disease [27,28]. During the year 2021, several veterinary directorates in different districts throughout Tanzania reported suspected outbreaks of ND. Due to the vast genomic diversity of NDV, diagnostic failures are more likely to occur, leading to infections going undetected, which could lead to larger outbreaks and greater economic losses. Our study, therefore, consisted in collecting samples in the areas concerned.

Previous studies found that genotypes V and XIII are the most common genotypes in Tanzania, with both genotypes being identified in various regions [29,30,31]. Moreover, six NDV genotypes have been identified in Tanzania: I, II, V, VII, XIII, and XX [29,30,32,33,34]. Backyard poultry farms (BPFs) contribute to the maintenance and transmission of NDVs. Thus, in order to improve disease surveillance in Tanzania, the current study aimed to assess the NDV genotypes circulating in BPFs.

## 2. Materials and Methods

### 2.1. Sampling

Cloacal or tracheal swabs were collected from 43 vaccinated and 19 unvaccinated chickens with suspected respiratory viral infections in six Tanzanian regions (Arusha, Dar es Salaam, Dodoma, Iringa, Morogoro and Rukwa) during 2021. The proventriculus, trachea, brain and liver of 17 dead chickens were collected upon postmortem examination to assess pathogenic lesions. A single-use virus specimen collection tube/viral transport tube with swab/Viral Transport Medium/Universal Transport Medium (Chengdu Rich Science Industry, Chengdu, China) was used to collect samples (n = 79), which were coded, frozen in liquid nitrogen in the field and taken to the SACIDS Africa Centre of Excellence for Infectious Diseases’ lab, where they were immediately stored at −80 °C until additional examination.

### 2.2. Virus Isolation

Virus isolation was carried out by inoculating into specific-pathogen-free (SPF) embryonated chicken eggs (ECEs), as previously described [35]. Sterile forceps and scissors were used to cut tissue samples into small pieces, which were ground with sterile sand in a mortar and pestle. These pieces were then transferred to sterile 15 mL Falcon tubes that contained phosphate-buffered saline (PBS) at pH 7.4, penicillin (1000 IU/mL), streptomycin (10 mg/mL), gentamycin (250 µg/mL), and mycostatin (1000 IU/mL). Samples were centrifuged at 3000 rpm at 4 °C for 10 min to clarify the homogenized tissue suspensions, which were then transferred to new sterile microcentrifuge tubes and used immediately or stored at −80 °C. Ten-day-old SPF ECEs were inoculated with 100 µL of a 1:100 dilution of the clarified tissue homogenate via the allantoic route. Four days of incubation at 37 °C and candling were conducted for all inoculated SPF ECEs. After detecting the death of an embryo or at the end of the incubation period in the eggs, all eggs were kept at 4 °C for at least two hours before harvesting the allantoic fluid (AF) [36], which was then stored at −80 °C.

### 2.3. Hemagglutination Assay (HA)

The HA was performed on the AF of isolated strains using 1% chicken red blood cells, as previously described [37]. Briefly, 50 µL of AF obtained from the inoculated SPF eggs was added in triplicate to a 96-well microtiter plate. To each well, 25 µL of 1% chicken red blood cell suspension was added, and the plate was incubated at room temperature for 30 min. The titer of the HA test was determined by identifying the highest dilution of the virus that resulted in agglutination of the chicken red blood cell suspension. Because HA activity can be caused by various avian viruses, PCR was performed to confirm the presence of NDV only in the HA-positive AF samples.

### 2.4. Viral RNA Extraction and Reverse-Transcription Polymerase Chain Reaction (RT-PCR)

The RNA from AF was extracted using the QIAamp viral RNA extraction kit (Qiagen^®^, Valencia, CA, USA), as directed by the manufacturer. Until further examination, the extracted RNA was kept at −80 °C. The HA-positive AFs were subjected to RT-PCR using the SuperScript III one-step RT-PCR system with Platinum Taq DNA polymerase (Thermo Fisher Scientific, Carlsbad, CA, USA) to amplify the partial *F* gene using previously reported primers [38]. The expected length of the amplification products was 535 bp. On 1.5% agarose gel stained with GelRed^®^ (Phenix, Hong Kong, China), the PCR products were electrophoresed.

### 2.5. DNA Sequencing, Genetic Analysis, and Phylogeny Reconstruction

Before the PCR product was ready for Sanger sequencing, the one-step Applied Biosystems™ ExoSAP-IT™ Express PCR Product Cleanup Reagent (Applied Biosystems, Foster City, CA, USA) was applied to our reaction mixtures according to the manufacturer’s protocol to prevent unincorporated primers and dNTPs from interfering with our results.

Sequencing reactions were performed in the DNA Master cycler pro 384 (Eppendorf) using the ABI BigDye^®^ Terminator v3.1 Cycle Sequencing Kit (Applied Biosystems), following the protocols supplied by the manufacturer. Single-pass sequencing was performed on each template using specific primers. The fluorescence-labeled fragments were purified from the unincorporated terminators with the BigDye XTerminator^®^ Purification Kit (Applied Biosystems). The samples were injected to electrophoresis in an ABI 3730xl DNA Analyzer (Applied Biosystems).

Geneious Prime 2023.0 was used to assess the quality of the chromatograms, assemble and edit the obtained nucleotide sequences. NCBI BLAST was used to compare the sequences with those found in the NCBI nucleotide database. MEGA X software was used for nucleotide alignments using the ClustalW method [39]. Phylogenetic analysis based on the *F* gene was performed using the maximum likelihood method with the general time-reversible (GTR) model with a discrete gamma distribution (+G), allowing for invariant sites (+I) and 1000 bootstrap resampling [39]. The graphical user interface Sequence Demarcation Tool Version 1.2 (SDTv1.2) (available at www.cbio.uct.ac.za/SDT) was used in the study to calculate the pairwise percentage nucleotide identity of each sequence [40]. The partial *F* gene sequences (n = 40) of NDV obtained in this study were submitted to GenBank and are available under the accession numbers ON148417 to ON148434, and OQ434700 to OQ434721.

## 3. Results

### 3.1. Clinical and Postmortem Findings

**Figure 1:**
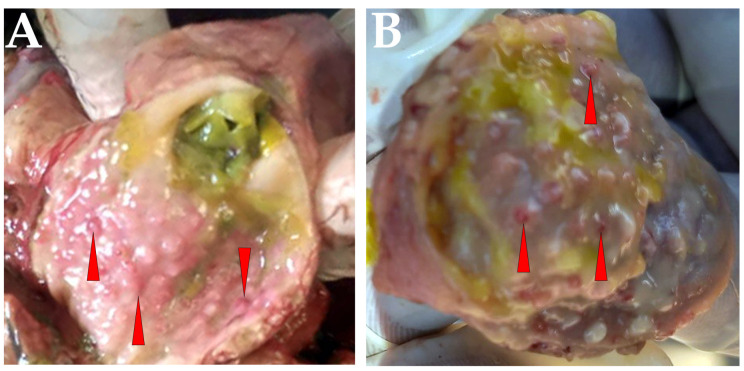
Clinical signs and necropsy findings in domestic chickens with Newcastle disease (ND). Chickens in a backyard poultry farm where suspected ND cases occurred, showing (**A**,**B**) pinpoint petechial hemorrhage on the proventriculus glands (indicated by a red arrowhead).

Various clinical signs were observed in chickens, including greenish diarrhea, coughing and runny nose, tremors and torticollis, watery eyes and swelling of the eyes. Signs of sepsis were also observed at autopsy, including hemorrhages at the ends of the proventriculus, congested blood vessels and greenish mucous contents in the gastrointestinal tract (Figure 1A,B).

### 3.2. Virus Isolation

We inoculated SPF ECEs with each sample. Within 48–96 h after inoculation, most of the embryos showed extensive hemorrhage, congestion, and death. Following the first passage, 57 out of 79 (72.2%) of harvested AF samples exhibited positive hemagglutination activity (Table 1). Three additional passages of negative AFs were performed to detect HA activity, but no additional positives were detected; these samples were classified as negative.

### 3.3. RT-PCR Screening for NDV

One-step RT-PCR for partial *F* gene amplification was used to screen the 57 HA-positive samples for NDV. We selected this region because it contains the multibasic cleavage site, which is a major determinant of virulence [41]. Our results revealed that 50 samples were positive for NDV (Supplementary Figure S1). Overall, 87.7% (50/57) of the HA-positive samples and 63.3% (50/79) of all samples collected were positive for NDV (Table 1).

### 3.4. Amino Acid Analyses

Given the funds available for further analysis, while ensuring that selected sequences were revealed from each of the regions covered by our study, 40 isolates out of the 50 NDV isolates were selected for partial *F* gene sequencing, as previously described [42]. Typically, virulent strains of NDV have two or more basic amino acids (arginine or lysine) located at positions 112 to 116 of the fusion protein and phenylalanine at position 117 [35], whereas lentogenic strains have only one basic amino acid at this site [43]. The deduced amino acid sequences of the fusion protein cleavage site (FPCS) for the isolates analyzed in this study revealed that 38 out of 40 isolates had the sequence 112-RRRKRF-117 and two had 112-RRQKRF-117, which indicates that all of the isolates are virulent [44] (Table 2).

### 3.5. Phylogenetic Analyses

**Figure 2:**
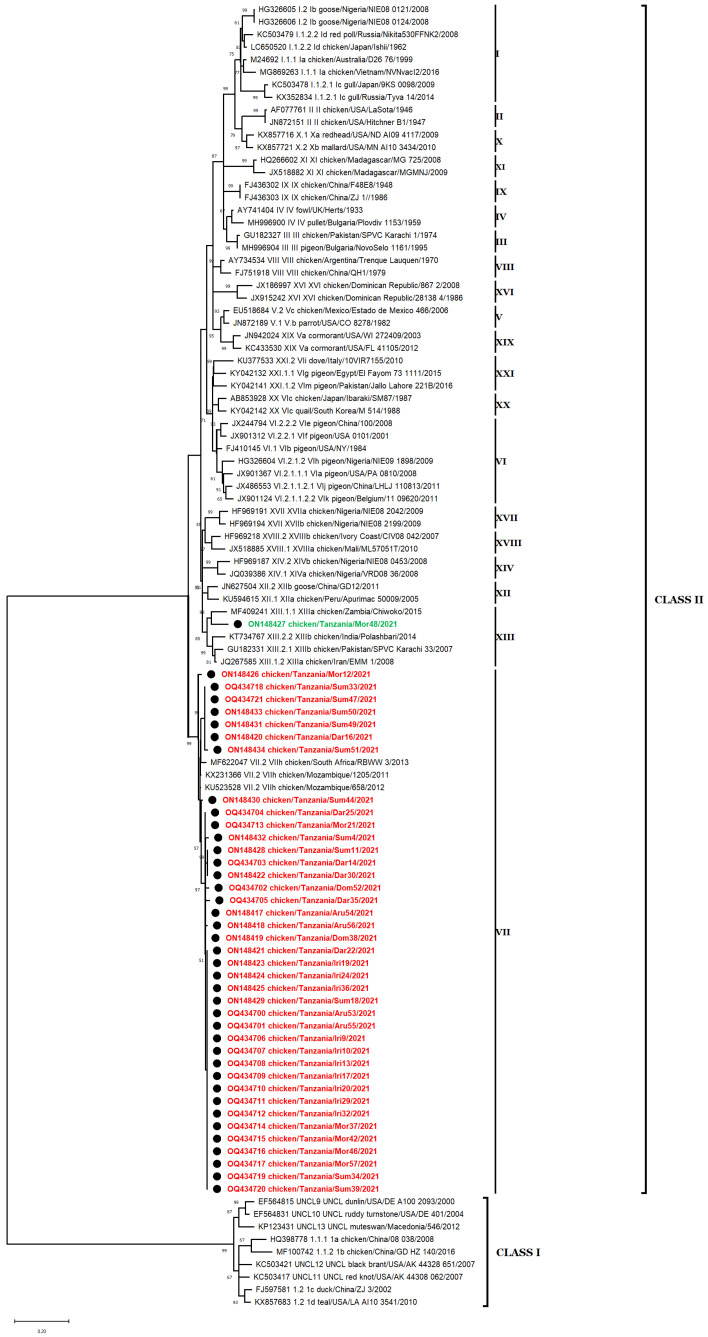
Phylogenetic analyses based on the partial *F* gene sequences of NDV strains. The 40 nucleotide sequences described in this study are marked with ● and labeled in red (subgenotype VII.2) or green (subgenotype XIII.1.1). Sixty-five nucleotide sequences representing all class I (including five unclassified viruses of class I, UNCL 9 to 13) and II subgenotypes were involved in this analysis. Phylogenic relationships using 1000 bootstraps were determined with MEGA X [39] using ClustalW alignment algorithm and maximum likelihood method for phylogenetic tree reconstruction. The evolutionary history was inferred by using the maximum likelihood and general time-reversible (GTR) model with a discrete gamma distribution (+G), allowing for invariant sites (+I) [45]. Codon positions included in the analysis were 1st + 2nd + 3rd + noncoding.

To discover the phylogenetic connection between the sequences described in this study and previously reported NDV isolates, representative sequences from each genotype [8] were used to determine the clustering of isolates obtained in this study. For this purpose, in addition to the 40 sequences reported in this study, 102 sequences of NDV class I and II isolates from NCBI BLAST were used. The pilot tree was used for rapid preliminary identification of the complete dataset of NDV sequences, allowing for the classification of the isolates according to their genotypes. It was found that all the 40 NDV isolates collected from backyard poultry farms in Tanzania belong to class II, with 39 of the isolates belonging to genotype VII and one isolate belonging to genotype XIII (Figure 2). The nucleotide similarity between the strains of this study and previously identified isolates provided information on the strains with the highest homology for our 39 subgenotype VII.2 strains and our single subgenotype XIII.1.1 strain (Supplementary Table S1).

### 3.6. Genotype VII Isolates

**Figure 3:**
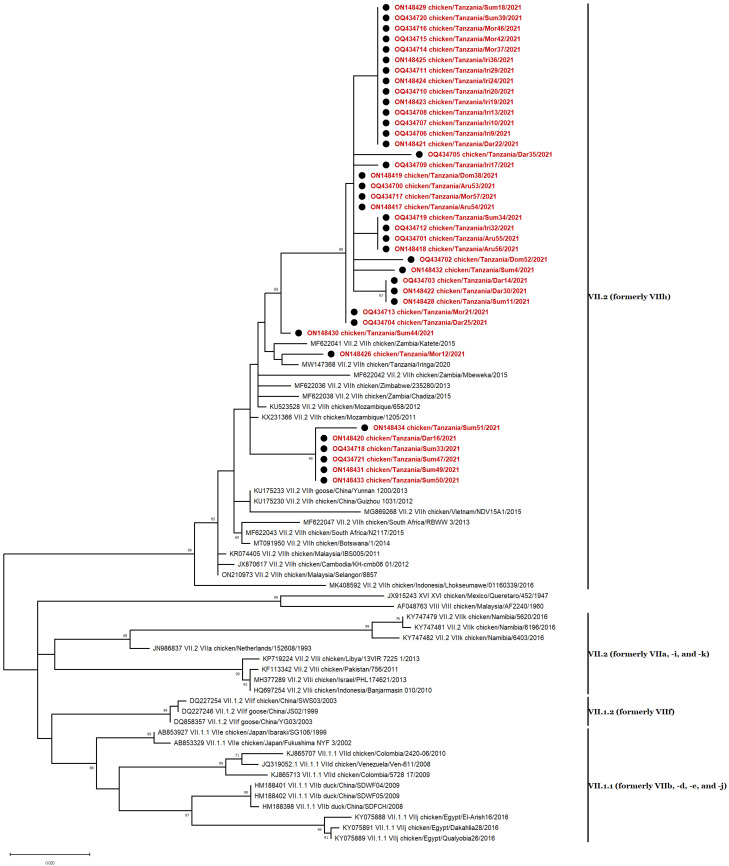
Phylogenetic analyses based on the partial *F* gene nucleotide sequences of NDV strains. The 39 genotype VII nucleotide sequences described in this study are marked with ● and labeled in red. Forty-one nucleotide sequences representing all genotype VII subgenotypes and 2 outliers (JX915243 from genotype XVI and AF048763 from genotype VIII) were included in this analysis. Phylogenic relationship using 1000 bootstraps was determined with MEGA X [39] using ClustalW alignment algorithm and maximum likelihood method for tree construction. The evolutionary history was inferred by using the maximum likelihood and general time-reversible (GTR) model with a discrete gamma distribution (+G), allowing for invariant sites (+I) [45]. Codon positions included in the analysis were 1st + 2nd + 3rd + noncoding.

The 39 NDV isolates of genotype VII were detected in Arusha (n = 4), Dar es Salaam (n = 6), Dodoma (n = 2), Iringa (n = 10), Morogoro (n = 6), and Sumbawanga (n = 7), Tanzania, and all belong to subgenotype VII.2, previously classified as genotype VIIh (Figure 3) [8,46]. Genotype VII sequences were closely related to strains previously found in Mozambique (KU523528, 2012), South Africa (MF622043, 2015), Zimbabwe (MF622036, 2013), and Tanzania (MW147368, 2020) [30,33,47,48]. The highest homology for our 39 subgenotype VII.2 is with a strain isolated from a broiler chicken in Mozambique in 2012 (KU523528) [48], sharing between 96.64% and 98.50% nucleotide identity with all isolates. These data suggest that while the isolates found here are related to previously identified strains, they also have a considerable range of divergence.

### 3.7. Genotype XIII Isolate

**Figure 4:**
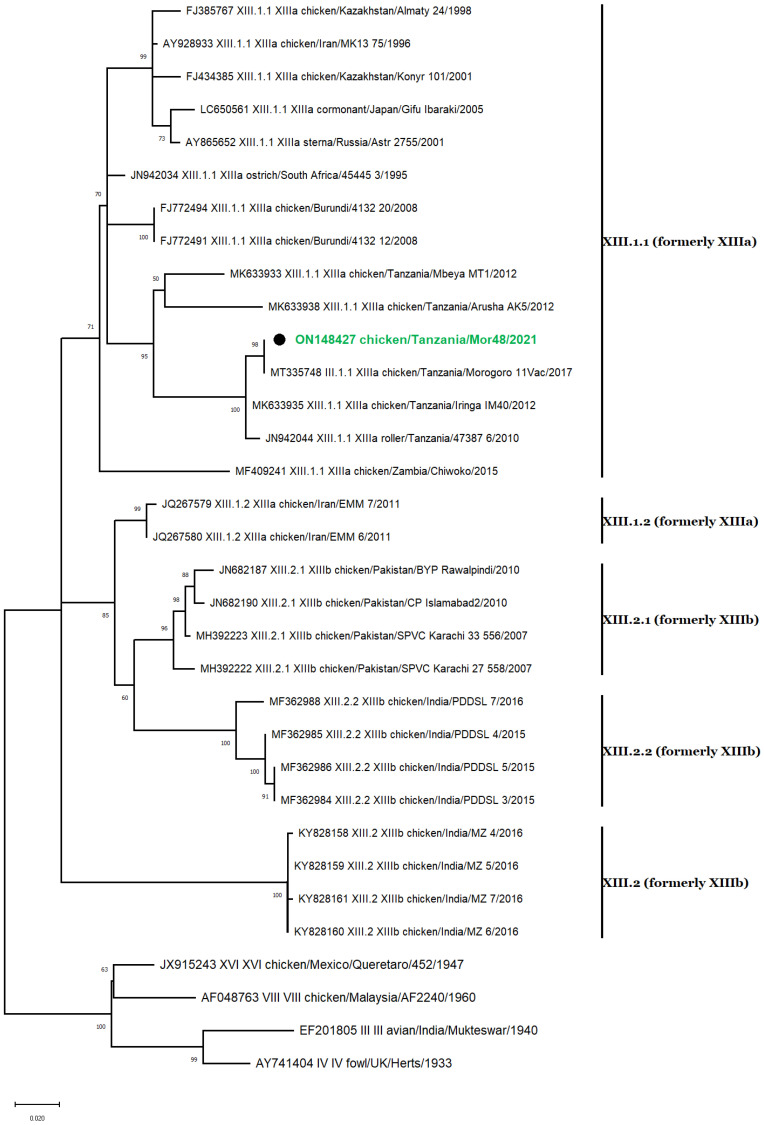
Phylogenetic analyses based on the partial *F* gene nucleotide sequences of NDV strains. The single genotype XIII nucleotide sequence described in this study is marked with ● and labeled green. Thirty-two nucleotide sequences representing all genotype XIII subgenotypes and 4 outliers (AY741404 from genotype IV, EF201805 from genotype III, JX915243 from genotype XVI, and AF048763 from genotype VIII) were included in this analysis. Phylogenic relationship using 1000 bootstraps was determined with MEGA X [39] using ClustalW alignment algorithm and maximum likelihood method for tree construction. The evolutionary history was inferred by using the maximum likelihood and general time-reversible (GTR) model with a discrete gamma distribution (+G), allowing for invariant sites (+I) [45]. Codon positions included in the analysis were 1st + 2nd + 3rd + noncoding.

The single NDV isolate of class II genotype XIII was collected from a chicken in the Morogoro region (Figure 4). The isolate clustered with strains that belonged to subgenotype XIII.1.1, previously known as XIIIa (Figure 4) [8,46] and was closely related to an isolate previously reported in Tanzania (MT335748) [30], sharing 99.44% nucleotide identity.

### 3.8. Nucleotide Sequence Similarity

**Figure 5:**
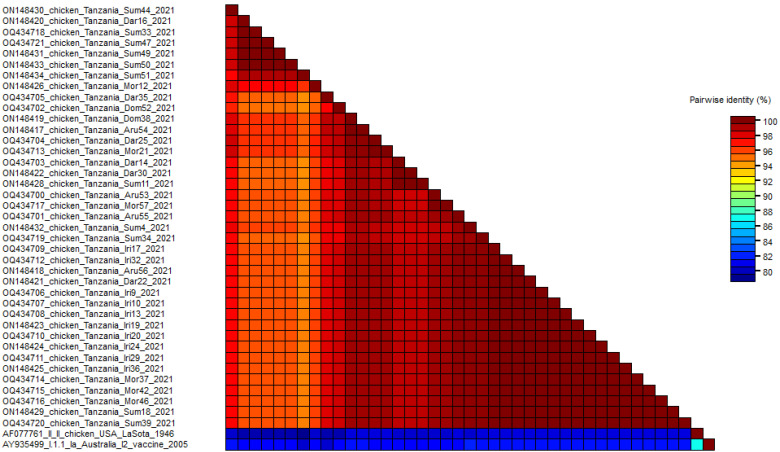
Pairwise identities of the 39 genotype VII strains’ partial fusion protein sequences that were aligned by ClustalW and shown by the software Sequence Demarcation Tool (SDT).

The nucleotide sequence identity of the 39 NDV genotype VII strains in this study ranged from 94% to 100% (Figure 5). The identity with the LaSota and I2 vaccine strains was 78% (Figure 5). All genotype VII isolates differed by 18–22% in amino acid composition from vaccines administered in Tanzania (LaSota [genotype II] and I2 [genotype I]).

**Figure 6:**
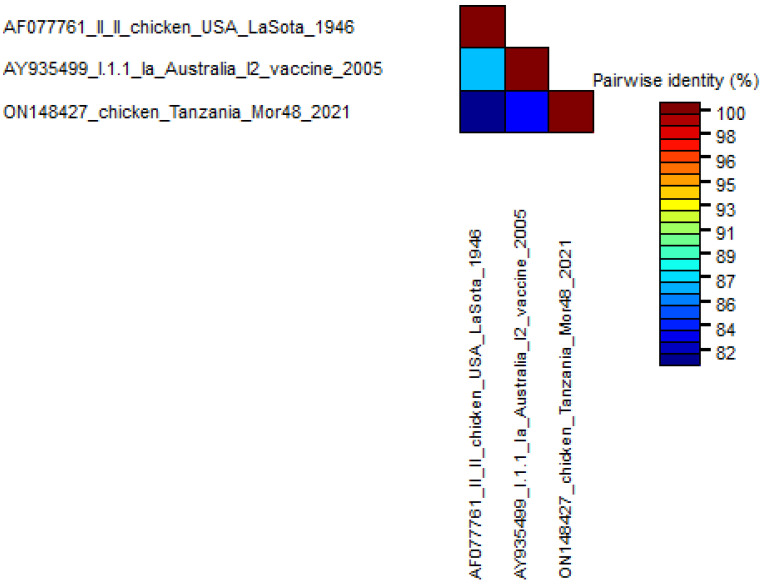
Sequence Demarcation Tool (SDT) software’s display of the pairwise identities plot of fusion protein sequences aligned by ClustalW for our sole genotype XIII strain.

According to Figure 6, the genotype XIII isolate differed by 13–18% in amino acids from the LaSota [genotype II] and I2 [genotype I] vaccines, which are frequently administered in Tanzania. In comparison to genotype VII strains, our genotype XIII isolate had a higher identity (87%) with the LaSota and I2 vaccine strain.

## 4. Discussion

Understanding the genetic diversity of viral pathogens is necessary for better control of endemic animal diseases in rural areas, which have major economic impacts [49]. Currently, there is little information on the circulating NDV genotypes within Tanzania, the 24th most populous country in the world and a major hub connecting Southern and Eastern Africa. Here, we collected samples from backyard poultry farms throughout Tanzania to assess the circulating genotypes of NDV. We collected 79 total samples from 16 locations and found 57 samples positive by HA test. The presence of NDV was detected in 50 out of 57 (87.7%) samples. Since all birds sampled had clear signs of disease, the seven NDV-negative samples may have been infected with other avian pathogens. An analysis of the additional samples could help to provide information on diseases coexisting with NDV on Tanzanian farms.

Based on sequence and phylogenetic analysis of a subset of 40 isolates, a single sample clustered in subgenotype XIII.1.1 with a closely related virus previously reported in Tanzania in 2017 [30]. The remaining 39 isolates belonged to subgenotype VII.2 and were closely related to isolates previously reported from Mozambique [48], South Africa [47], and Tanzania [33]. The NDV subgenotype XIII.1.1 isolate we identified in this study was isolated from the Morogoro region, where it had recently been identified [30]. Subgenotype XIII.1.1 is considered virulent and has caused outbreaks in Asia, Europe and Africa [19]. The viscerotropic nature of NDV subgenotype XIII.1.1 was confirmed through the analysis of its clinicopathological features in a recent study [24]. Subgenotype XIII.1.1 has also been implicated in ND outbreaks in other African countries, namely, Zambia [50], Burundi and South Africa [51]. Outbreaks of genotype XIII virus have been reported in vaccinated farms in Bangladesh and India. This may suggest that the vaccine strains have poor efficacy against this genotype [18,24,52,53].

In Tanzania, genotype VII.2 was first identified in the Mwanza region (northwest Tanzania) in 2012 [30], and was more recently identified in 2020 in local chickens in the Iringa region (southwest Tanzania) [33]. The results of our study reveal that genotype VII is now dominant in the regions of Tanzania where we sampled. The dispersal of subgenotype VII.2 throughout the studied areas could be explained by vaccination failure and bad management practices; for example, poultry trading between Tanzania and Mozambique may explain the wide circulation of this genotype in Tanzania [31], which was first found in Mozambique in 2011 [47]. After being detected for the first time in Africa in 2011, especially in Mozambique, where it caused outbreaks in vaccinated broilers, subgenotype VII.2 has already been detected in vaccinated chicken farms in countries such as Malawi, Zambia, Zimbabwe, South Africa and Botswana [47,54,55,56].

To date, six NDV genotypes have been identified in Tanzania: I, II, V, VII (including subgenotype VII.2), XIII, and XX. Genotypes V and XIII have been reported as the most common genotypes in Tanzania for decades and have been identified in different parts of the country [29,30,31,32,34]. Given that the two most virulent NDV genotypes to date remain VII and XIII [17,57], the results of this study are troubling.

This study also highlights the importance of understanding NDV evolutionary dynamics in Tanzania. It is important to monitor the rich avian biodiversity of Tanzania, since widespread NDV infection presents a potential opportunity for virulent strains of NDV to emerge and spread throughout southeast Africa. In previous studies, NDV strains circulating in Tanzania have been investigated in both commercial (MK583011; MK633932-MK633963) [31,32], and in backyard poultry farms (MT335727-MT335750) [30]. The possible spread of NDV across different types of farm, namely, backyard and commercial farms, had already been highlighted by Yongolo et al. [34], who had shown from their analysis that NDV was persistently present among chicken populations and possibly spread through live chicken markets or wild bird migration. It should be remembered that a potential risk factor for ND transmission to commercial farms in China has been identified as a high reported seroprevalence of NDV in backyard poultry [58] and southern Brazil [59]. The proximity of commercial poultry to backyard poultry in rural Africa is a reality, and there is a risk that genotype VII will expand and have a greater impact on poultry farming and food security in general in Tanzania. The similarity of virus characteristics circulating in backyard and commercial chickens in Tanzania was also demonstrated by the detection in the Morogoro region of an isolate (ON148427) grouped with strains belonging to subgenotype XIII.1.1 and closely related to an isolate previously reported in broilers in the same region (MT335748) [30], sharing 99.44% nucleotide identity.

As vaccination is the most effective way to prevent Newcastle disease in poultry, it should be underlined that the most common vaccines against ND in Tanzania are the LaSota and I-2 live virus vaccines. It was previously reported that 58% of Tanzanian households are using I-2 and 42% are using LaSota [60]. The I-2 strain was recommended for ND vaccination in hot climates since it minimizes temperature-induced degradation [61]. As recently suggested by Dimitrov [1], the efficacy of current live attenuated vaccines against currently circulating strains of NDV and vaccine application failure should be considered when designing new, more effective vaccines. Thus, in addition to the thermotolerance and mode of administration already challenging the efficacy of the I-2 and LaSota vaccines, the genetic relationship between these commercially available vaccines and the Tanzanian isolates might explain the ongoing ND outbreaks. It may make sense to develop genotype-matched vaccines to help control ND.

## 5. Conclusions

In view of the problems posed by genotypes VII and XIII elsewhere, regular monitoring and characterization of circulating strains, as well as expansion of the study to other regions of southeast Africa will provide a more accurate picture of the ND situation and contribute to improved disease control. These results also suggest the possibility that current vaccines do not prevent infection caused by virulent strains of NDV in Tanzania. Therefore, better genotype-matched vaccines, along with improved biosafety practices, could help reduce the economic losses caused by ND.

## Figures and Tables

**Table 1:** Results of one-step PCR and hemagglutination assay.

Zone	Regions	Number of Samples	Test	
			HA	RT-PCR
			Positive	Positive
Northern	Arusha	7	6	6
Coastal	Dar es Salaam	9	8	7
Coastal	Morogoro	8	7	7
Central	Dodoma	16	7	2
Southern Highlands	Iringa	12	12	12
Southern Highlands	Rukwa	27	17	16
Total		79	57/79	50/79

**Table 2:** An overview of the NDV isolates isolated in this study.

GenBank Accession	Isolate ID	Subgenotype	Location	Collection Date	Sample Type	Vaccination Status	Vaccine	Cleavage Site Motif ^b^	Pathotype
OQ434700	Aru53	VII.2	Arusha	15 October 2021	Tissue	Vaccinated	LaSota	RRRKRF	Virulent (velogenic)
ON148417	Aru54	VII.2	Arusha	15 October 2021	Swab	Unvaccinated	N/A ^a^	RRRKRF	Virulent (velogenic)
OQ434701	Aru55	VII.2	Arusha	15 October 2021	Tissue	Vaccinated	I-2	RRRKRF	Virulent (velogenic)
ON148418	Aru56	VII.2	Arusha	15 October 2021	Swab	Unvaccinated	N/A	RRRKRF	Virulent (velogenic)
ON148419	Dom38	VII.2	Dodoma	18 November 2021	Swab	Unvaccinated	N/A	RRRKRF	Virulent (velogenic)
OQ434702	Dom52	VII.2	Dodoma	19 August 2021	Tissue	Vaccinated	LaSota	RRRKRF	Virulent (velogenic)
OQ434703	Dar14	VII.2	Dar es Salaam	19 August 2021	Swab	Unvaccinated	N/A	RRRKRF	Virulent (velogenic)
ON148420	Dar16	VII.2	Dar es Salaam	19 August 2021	Swab	Unvaccinated	N/A	RRRKRF	Virulent (velogenic)
ON148421	Dar22	VII.2	Dar es Salaam	29 August 2021	Swab	Unvaccinated	N/A	RRRKRF	Virulent (velogenic)
OQ434704	Dar25	VII.2	Dar es Salaam	29 August 2021	Swab	Vaccinated	LaSota	RRRKRF	Virulent (velogenic)
ON148422	Dar30	VII.2	Dar es Salaam	4 September 2021	Swab	Vaccinated	I-2	RRRKRF	Virulent (velogenic)
OQ434705	Dar35	VII.2	Dar es Salaam	4 September 2021	Tissue	Vaccinated	I-2	RRRKRF	Virulent (velogenic)
OQ434706	Iri9	VII.2	Iringa	14 July 2021	Tissue	Vaccinated	I-2	RRRKRF	Virulent (velogenic)
OQ434707	Iri10	VII.2	Iringa	14 July 2021	Swab	Unvaccinated	N/A	RRRKRF	Virulent (velogenic)
OQ434708	Iri13	VII.2	Iringa	14 July 2021	Swab	Unvaccinated	N/A	RRRKRF	Virulent (velogenic)
OQ434709	Iri17	VII.2	Iringa	14 July 2021	Tissue	Vaccinated	I-2	RRRKRF	Virulent (velogenic)
ON148423	Iri19	VII.2	Iringa	23 August 2021	Swab	Vaccinated	LaSota	RRRKRF	Virulent (velogenic)
OQ434710	Iri20	VII.2	Iringa	23 August 2021	Swab	Vaccinated	LaSota	RRRKRF	Virulent (velogenic)
ON148424	Iri24	VII.2	Iringa	23 August 2021	Swab	Vaccinated	LaSota	RRRKRF	Virulent (velogenic)
OQ434711	Iri29	VII.2	Iringa	23 August 2021	Swab	Vaccinated	LaSota	RRRKRF	Virulent (velogenic)
OQ434712	Iri32	VII.2	Iringa	23 August 2021	Swab	Vaccinated	LaSota	RRRKRF	Virulent (velogenic)
ON148425	Iri36	VII.2	Iringa	23 August 2021	Swab	Unvaccinated	N/A	RRRKRF	Virulent (velogenic)
ON148426	Mor12	VII.2	Morogoro	2 October 2021	Tissue	Vaccinated	LaSota	RRQKRF	Virulent (velogenic)
OQ434713	Mor21	VII.2	Morogoro	2 October 2021	Swab	Vaccinated	LaSota	RRRKRF	Virulent (velogenic)
OQ434714	Mor37	VII.2	Morogoro	2 October 2021	Swab	Vaccinated	I-2	RRRKRF	Virulent (velogenic)
OQ434715	Mor42	VII.2	Morogoro	2 October 2021	Swab	Unvaccinated	N/A	RRRKRF	Virulent (velogenic)
OQ434716	Mor46	VII.2	Morogoro	2 October 2021	Swab	Unvaccinated	N/A	RRRKRF	Virulent (velogenic)
ON148427	Mor48	XIII.1.1	Morogoro	2 October 2021	Swab	Vaccinated	LaSota	RRQKRF	Virulent (velogenic)
OQ434717	Mor57	VII.2	Morogoro	9 October 2021	Tissue	Vaccinated	LaSota	RRRKRF	Virulent (velogenic)
ON148432	Sum4	VII.2	Sumbawanga	25 July 2021	Swab	Unvaccinated	N/A	RRRKRF	Virulent (velogenic)
ON148428	Sum11	VII.2	Sumbawanga	21 June 2021	Tissue	Vaccinated	LaSota	RRRKRF	Virulent (velogenic)
ON148429	Sum18	VII.2	Sumbawanga	21 June 2021	Tissue	Vaccinated	LaSota	RRRKRF	Virulent (velogenic)
OQ434718	Sum33	VII.2	Sumbawanga	22 June 2021	Swab	Unvaccinated	N/A	RRRKRF	Virulent (velogenic)
OQ434719	Sum34	VII.2	Sumbawanga	22 June 2021	Swab	Vaccinated	I-2	RRRKRF	Virulent (velogenic)
OQ434720	Sum39	VII.2	Sumbawanga	22 June 2021	Swab	Vaccinated	LaSota	RRRKRF	Virulent (velogenic)
ON148430	Sum44	VII.2	Sumbawanga	22 June 2021	Swab	Unvaccinated	N/A	RRRKRF	Virulent (velogenic)
OQ434721	Sum47	VII.2	Sumbawanga	22 June 2021	Swab	Unvaccinated	N/A	RRRKRF	Virulent (velogenic)
ON148431	Sum49	VII.2	Sumbawanga	22 June 2021	Tissue	Vaccinated	LaSota	RRRKRF	Virulent (velogenic)
ON148433	Sum50	VII.2	Sumbawanga	23 June 2021	Tissue	Vaccinated	LaSota	RRRKRF	Virulent (velogenic)
ON148434	Sum51	VII.2	Sumbawanga	27 June 2021	Tissue	Vaccinated	LaSota	RRRKRF	Virulent (velogenic)

^a ^N/A not applicable; ^b^ F-protein cleavage site motif (112–117).

## Data Availability

All data generated in the present study are available in the published manuscript and its Supplementary Materials.

## Supplementary Materials

The following supporting information can be downloaded at https://www.mdpi.com/article/10.3390/vetsci10070477/s1. Figure S1: Gel electrophoresis of the amplified fusion gene of field isolates. The positive sample is 535 base pairs (bp) in size, lane M 100-bp DNA ladder, lane 1–57 tested sample identification numbers, lane PC known positive control sample, lane NC negative control (RNase free water), Table S1: Comparison of NDV strains from the 2021 outbreaks in Tanzania and previously reported strains retrieved from NCBI GenBank.
