# Supplementary material for: Genetic Diversity of Newcastle Disease Virus Involved in the 2021 Outbreaks in Backyard Poultry Farms in Tanzania
Source: Vet Sci. 2023 Jul 21;10(7):477. doi: 10.3390/vetsci10070477 (PMC10385779; doi:10.3390/vetsci10070477)
Supplement: Supplementary file 1 [file vetsci-10-00477-s001.zip › vetsci-2416769-supplementary.pdf]

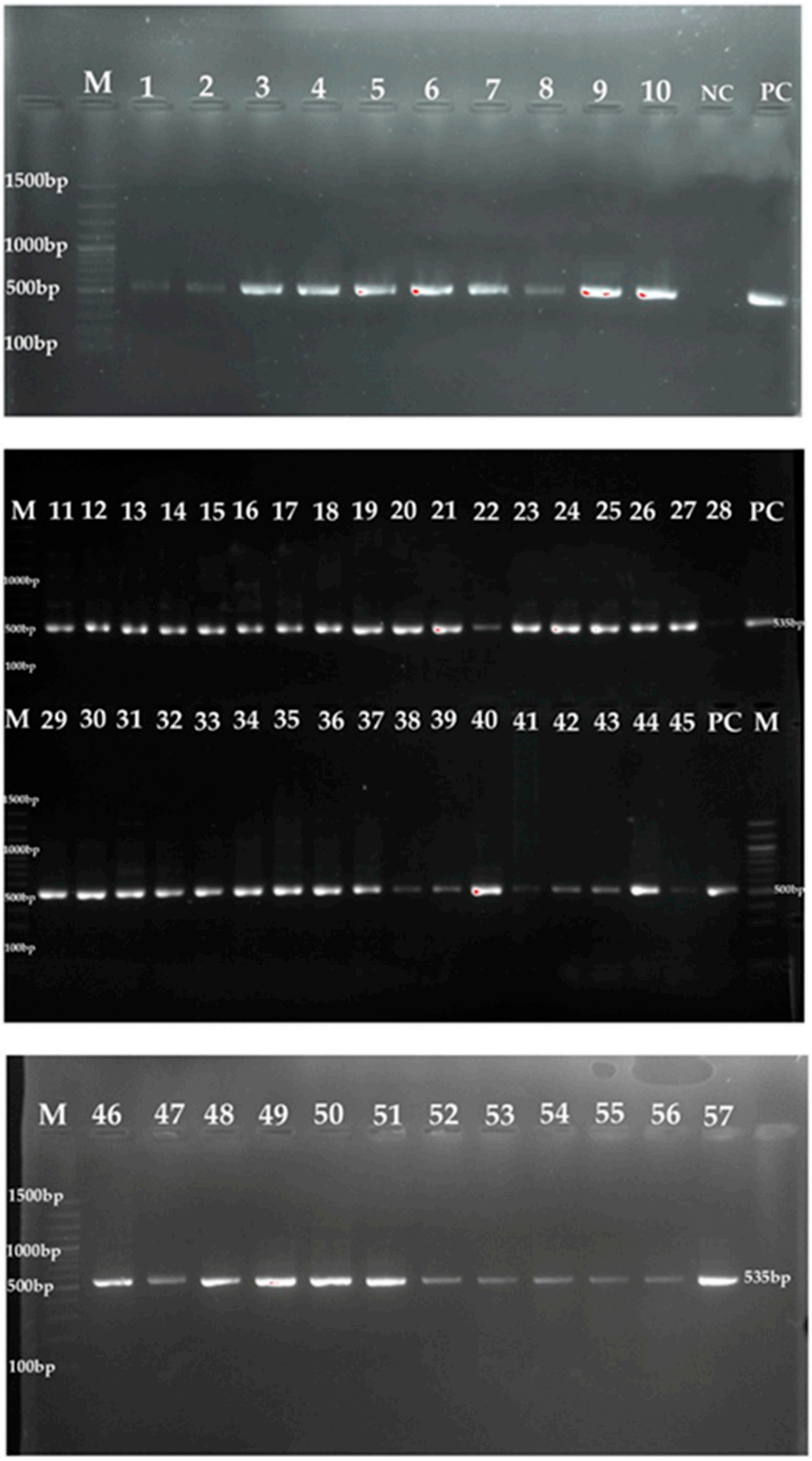

Figure S1. Gel electrophoresis of the amplified fusion gene of field isolates. The positive sample is 535 base pairs (bp) in size, Lane M 100-bp DNA Ladder, Lane 1-57 tested sample identification numbers, Lane PC known positive control sample, Lane NC Negative control (RNase free water)

Table S1. Comparison of NDV strains from the 2021 outbreaks in Tanzania and previously reported strains retrieved from NCBI GenBank.

GenBank accession #	Isolate ID	highest similar strain name	similar strain accession	percent identity	Similar strain sub-genotype
OQ434700	Aru53	NDV/chicken/Mozambique/658/2012	KU523528	97.20%	VII.2
ON148417	Aru54	NDV/chicken/Mozambique/658/2012	KU523528	97.76%	VII.2
OQ434701	Aru55	NDV/chicken/Mozambique/658/2012	KU523528	96.82%	VII.2
ON148418	Aru56	NDV/chicken/Mozambique/658/2012	KU523528	98.13%	VII.2
ON148419	Dom38	NDV/chicken/Tanzania/Iringa/2020	MW147368	98.13%	VII.2
OQ434702	Dom52	NDV/chicken/Mozambique/658/2012	KU523528	96.07%	VII.2
OQ434703	Dar14	NDV/chicken/Mozambique/658/2012	KU523528	96.45%	VII.2
ON148420	Dar16	NDV/chicken/Tanzania/Iringa/2020	MW147368	98.50%	VII.2
ON148421	Dar22	NDV/chicken/Mozambique/658/2012	KU523528	98.32%	VII.2
OQ434704	Dar25	NDV/chicken/Mozambique/658/2012	KU523528	97.38%	VII.2
ON148422	Dar30	NDV/chicken/Mozambique/658/2012	KU523528	96.82%	VII.2
OQ434705	Dar35	NDV/chicken/Tanzania/Iringa/2020	MW147368	96.26%	VII.2
OQ434706	Ir9	NDV/chicken/Mozambique/658/2012	KU523528	96.64%	VII.2
OQ434707	Ir10	NDV/chicken/Mozambique/658/2012	KU523528	96.64%	VII.2
OQ434708	Ir13	NDV/chicken/Mozambique/658/2012	KU523528	96.64%	VII.2
OQ434709	Ir17	NDV/chicken/Mozambique/658/2012	KU523528	96.64%	VII.2
ON148423	Iri19	NDV/chicken/Mozambique/658/2012	KU523528	97.94%	VII.2
OQ434710	Ir20	NDV/chicken/Mozambique/658/2012	KU523528	96.64%	VII.2
ON148424	Iri24	NDV/chicken/Mozambique/658/2012	KU523528	97.38%	VII.2
OQ434711	Ir29	NDV/chicken/Mozambique/658/2012	KU523528	96.64%	VII.2
OQ434712	Ir32	NDV/chicken/Mozambique/658/2012	KU523528	96.64%	VII.2
ON148425	Iri36	NDV/chicken/Mozambique/658/2012	KU523528	97.20%	VII.2
ON148426	Mor12	NDV/chicken/Tanzania/Iringa/2020	MW147368	98.88%	VII.2
OQ434713	Mor21	NDV/chicken/Mozambique/658/2012	KU523528	97.38%	VII.2
OQ434714	Mor37	NDV/chicken/Mozambique/658/2012	KU523528	96.64%	VII.2
OQ434715	Mor42	NDV/chicken/Mozambique/658/2012	KU523528	96.64%	VII.2
OQ434716	Mor46	NDV/chicken/Mozambique/658/2012	KU523528	96.64%	VII.2
ON148427	Mor48	11Vac (Tanzania)	MT335748	99.44%	XIII.1.1
OQ434717	Mor57	NDV/chicken/Mozambique/658/2012	KU523528	96.64%	VII.2
ON148432	Sum4	NDV/chicken/Mozambique/658/2012	KU523528	98.50%	VII.2
ON148428	Sum11	NDV/chicken/Mozambique/658/2012	KU523528	97.01%	VII.2
ON148429	Sum18	NDV/chicken/Mozambique/658/2012	KU523528	96.64%	VII.2
OQ434718	Sum33	NDV/chicken/Tanzania/Iringa/2020	MW147368	98.13%	VII.2
OQ434719	Sum34	NDV/chicken/Mozambique/658/2012	KU523528	96.82%	VII.2
OQ434720	Sum39	NDV/chicken/Mozambique/658/2012	KU523528	96.64%	VII.2
ON148430	Sum44	NDV/chicken/Tanzania/Iringa/2020	MW147368	99.44%	VII.2
OQ434721	Sum47	NDV/chicken/Tanzania/Iringa/2020	MW147368	98.13%	VII.2
ON148431	Sum49	NDV/chicken/Mozambique/1205/2011	KX231366	98.67%	VII.2
ON148433	Sum50	chicken/South Africa/RBNW-1/2013	MF622045	97.57%	VII.2

ON148434	Sum51	chicken/South Africa/RBNW-1/2013	MF622045	98.95%	VII.2
